# Evidence-biased medicine? Applying a health equity lens to Sackett’s methodological framework

**DOI:** 10.3399/BJGP.2025.0385

**Published:** 2025-12-01

**Authors:** Caroline Mitchell, Teresa Hagan, Kate Fryer, Josephine Reynolds, Rebecca Mawson, Qizhi Huang, Laura Emery, Benjamin Duke, Mahendra G Patel

**Affiliations:** 1 School of Medicine, Keele University, Newcastle-under-Lyme, UK; 2 University of Sheffield, Sheffield, UK; 3 School of Medicine and Population Health, University of Sheffield, Sheffield, UK; 4 School of Medicine and Population Health, University of Sheffield, Sheffield, UK; 5 School of Medicine and Population Health, University of Sheffield, Sheffield, UK; 6 Centre for Research Equity, University of Oxford, Oxford, UK

## The inverse research law

The ‘inverse care law’ highlights how those who need health care the most often receive the least. This includes socioeconomically disadvantaged individuals, racially minoritised groups, people with disabilities, and other marginalised communities. These inequalities are exacerbated by the intersectionality of the social determinants and economic factors that shape health outcomes, and the systemic barriers that continue to limit access to equitable care.^1^ Expanding on this, the ‘inverse research law’ refers to the under-representation of these same underserved groups in medical research, even though they face a higher risk of ill health. Evidence-based medicine, the backbone of modern clinical practice, is only as good as the evidence it is derived from. If research representation does not truly reflect the diversity of the general population, medicines and other healthcare interventions may not work for everyone.^2^ Tackling the ‘inverse research law’ isn’t only about health justice, it improves health care for all by creating more rigorous science.

Sackett, the ‘father of evidence-based medicine’, described *bias* as: *‘any process at any stage of inference which tends to produce results or conclusions that differ systematically from the truth’.* ^3^ Bias is defined by the *Oxford Dictionary* as: *‘an inclination or prejudice for or against one person or group, especially in a way considered to be unfair’* and *‘a concentration on an interest in one particular area or subject’.* ^4^ When biases, whether political, institutional, or researcher driven, are embedded in medical research, they can become deeply rooted in clinical practice, perpetuate health disparities, and indirectly drive up healthcare costs through, for example, differential access to preventive measures, delayed diagnoses, and lack of treatment options.^5,6^ Gaps in understanding health conditions, and treatment effectiveness across ‘real-world’ diverse populations, pose a challenge in day-to-day health care where clinicians assume that their clinical practice is evidenced-*based,* rather than evidence-*biased,* and derived from sound scientific research. There are many examples of evidence-*biased* medicine, from medications that are less effective in certain groups, to racial profiling in pain management.^7,8^ Addressing these gaps is crucial to ensure that all populations benefit equally from evidence-based medicine.

Public trust in the recommendations of clinicians and research institutions is increasingly fragile, with vaccine hesitancy just one aspect of wider scepticism in medical science.^9^ The US, with its huge population and esteemed research institutions, is the global engine room of evidence-based medicine. Systematic undermining of the conceptual framework of our understanding of bias in research may dismantle the progress made in the reporting of key sociodemographic and ethnicity characteristics within clinical trials in the US and legitimise convenience sampling of more highly literate global-minority participants in research.^10^ Meanwhile, the UK National Institute for Health and Care Research (NIHR) has recently put research inclusion at the heart of its programmes by building research capacity in under-represented groups and mandating that NIHR-funded research underpins evidence-based medicine for all.

## Bias, positionality, and reflective research practice

Improving representation in research requires active efforts to break down systemic barriers and address mistrust caused by past injustices.^11,12^ The starting point of addressing bias in research is to consider the positionality of the researcher and the context: the interpretive, socially patterned culture and values of the research team and its institutions. Positionality refers to the social and political context that shapes the identity of the researcher, encompassing factors such as race, gender, class, and differential institutional power.^12^ This concept is crucial for understanding how a researcher’s background influences their perspective and potential biases in the research process.

Researchers, their institutions, and research funding bodies wield significant power in defining research problems and priorities, and in deciding who participates in and conducts the research. This power dynamic leaves some academic disciplines, women, and racialised minorities under-represented in senior roles. Dysfunctional institutional and disciplinary hierarchies persist, which limit opportunities for under-represented researchers to lead. The contextual ‘layers of privilege in the ivory tower of academia’ and enduring homogeneity within research teams might just as easily stifle creative vigour and innovation, as build world-leading science.^13^

Patient and public involvement and engagement in research (PPIE) has the potential to transform research by ensuring that it reflects the needs of patients and communities, and as a progressive intervention to challenge researcher positionality and associated biases across the research cycle. However, PPIE is itself characterised by a lack of sociocultural diversity and differential health literacy. PPIE representatives have described their involvement as tokenistic, with power and influence on the whole research cycle rarely ceded by academics and their institutions.^14^ By improving scientific communication, embracing diversity, genuinely sharing power, and democratising research, researchers can conduct more inclusive and impactful studies that enhance health and research literacy in underserved communities. This collaborative approach better serves both patients and researchers.

Reflective research practice, also known as reflexivity, involves a continuous process of self-awareness and critical analysis of one’s positionality and its impact on research study design, outputs, and fellow team members. Reflexivity is embedded in qualitative research methodology but rarely considered by quantitative researchers.^15^ We argue that reflective research practice should be normalised across the academic sector, supported by an institutional culture that embraces critical dialogue and champions equity. This embedded equity and team-focused scholarship is in stark contrast with tick-box ‘Diversity, Equity, and Inclusion’ activities.

## Research equity and Sackett’s Catalogue of Biases

**Figure 1.**
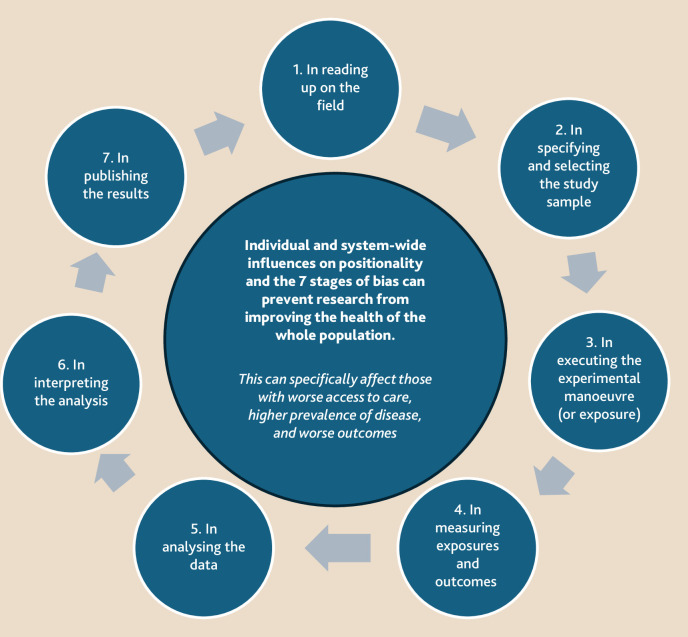
Equity-focused 7-step framework of bias (derived from Sackett: Appendix A — ‘Catalogue of Biases’).^3^

Sackett’s influential 1979 paper ‘Bias in Analytic Research’ was the first to systematically describe multiple forms of bias in clinical studies.^3^ In Appendix A of the paper, he outlined a 7-stage cycle whereby researchers, research teams, institutions, and the sociopolitical context of research culture can introduce bias into healthcare research, followed by 50+ referenced examples of bias (for example, ‘hot stuff’ bias, ‘obsequiousness’ bias, ‘data-dredging’ — ‘looking for the pony’ bias, ‘joggers’ bias). Applying an equity lens to Sackett’s framework highlights the issues that contribute to a self-perpetuating cycle of exclusion from research for underserved populations across multiple methodologies. Considering each step of this framework illustrates how the absence of reflection on positionality and societal accountability, and the sociopolitical and institutional influences on academia, might impact the validity, reliability, credibility, and generalisability of research findings (Figure 1).^3^

**Figure 2.**
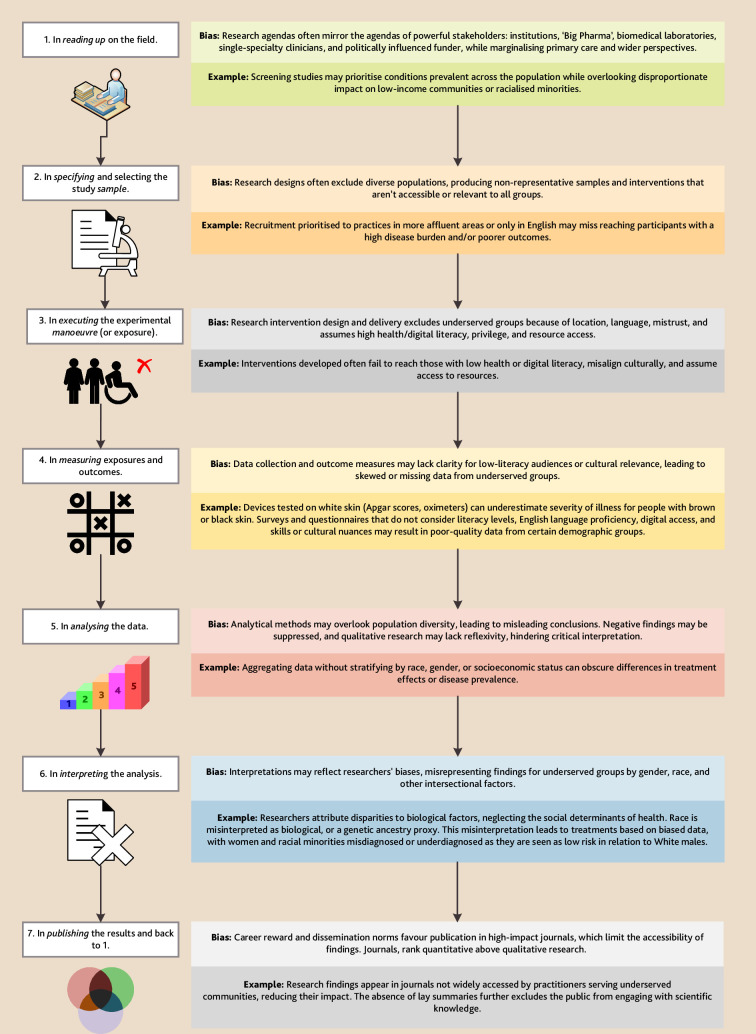
Equity bias in healthcare research with examples (adapted from Sackett’s framework of bias.^3^

However, researchers can identify and mitigate equity bias at each stage of the research cycle (Figure 2).

Sackett nominated several research priorities 50 years ago including: *‘The continued development of an annotated catalogue of bias. Each citation should include a useful definition, a referenced example illustrating the magnitude and direction of its effects, and a description of the appropriate preventive measures.’* ^3^

The Catalogue of Bias, maintained by the University of Oxford’s Centre for Evidence-Based Medicine, is an open-access, collaborative resource that builds on Sackett’s influential work to identify and explain biases that can distort health research.^16^ The Catalogue provides definitions, examples, and strategies to help researchers recognise, understand, and reduce bias in the design and interpretation of studies. It includes both well-known and lesser-known biases, aiming to improve the quality, transparency, and reliability of health research.

To address equity bias in research, we propose considering a Catalogue of Research Equity Bias based on the nine protected characteristics defined in UK and EU equality laws, and on constitutionally protected characteristics in the US and other global democracies.^17^ While the UK and EU consolidate these characteristics under laws such as the Equality Act 2010, US protections are addressed by various federal laws but are not unified under a single term. This contextual framework should also account for intersectionality and socially or medically patterned assumptions (Box 1). A Catalogue of Research Equity Bias, collaboratively developed by the research community, would be useful as an iterative reflective tool to assist individuals and research teams of all disciplines, healthcare specialties, and methodologies to address research equity bias in training, teamwork, and study design. The Oxford Catalogue of Bias features ‘Racial Bias’ as an exemplar of this approach.^16^

Box 1. **Protected characteristics 2021 (Equality and Human Rights Commission) with examples of research equity bias**

- Age^18^
- Disability: *includes neurodiversity, as well as multiple long-term conditions, mental and physical-related disability* ^19^
- Gender reassignment^20^
- Marriage and civil partnership
- Pregnancy and maternity^21^
- Race:^16^ *racial/ancestry assumptions bias, ethnicity data reporting/participation bias*
- Religion or belief
- Sex: gender^21,22^
- Sexual orientation^23^

**Examples of cross-cutting and intersectional research equity bias**

- Positionality bias: *convenience sampling bias* — a sample disproportionately reflects the characteristics of Eurocentric research teams, White ethnicity, higher socioeconomic status, rather than reflecting the population where disease burden is highest^24^
- Publication research equity bias: *novelty/hot-topic bias, ‘Big Pharma’ bias* ^25^
- Social class bias: constructs of social capital and social networks impact team composition and recruitment: *social class participation/data-reporting bias*
- Health literacy bias: over-technical communication by researchers, literacy requirements of recruitment materials, or interventions block participation
- Stigmatising bias*:* assumptions that marginalised communities are less reliable/deserving of research and health resources, for example, substance users, prisoners
- Specialty database bias: single-specialty silo perspectives exclude those with multiple long-term conditions and primary care populations; focusing on interventions for early-stage disease excludes underserved patients who present with later-stage disease

## Conclusion

This paper explores the role of academia in research justice, the crucial role of self- and team-aware reflective research practice and highlights the compromises to scientific quality and hence evidence-based medicine that occur if we allow the ‘inverse research law’ to prevail. By systematically addressing research equity bias, the medical community can work towards a more equitable healthcare system that provides effective and evidence-based care for all populations, particularly those who have been historically underserved.

We propose a solutions-oriented, low-resource call to action:

Research institutions and leaders should integrate reflective research practice, including consideration of research equity bias, into research culture and training programmes to challenge social norms and structures that foster poor scientific practices, non-inclusive teams, and, in some cases, research misconduct.

Medical journals should assess research equity bias in all submitted manuscripts and require authors to address equity considerations within the strengths and limitations section. An accessible online lay summary of the abstract should be published alongside the article, including a statement on bias and the research’s strengths and limitations.

Funding bodies should incorporate considerations of health and research equity bias into grant applications and into final reporting requirements.
